# Introjected regulation is the primary predictor of gaming disorder symptoms across WHO and APA criteria in a representative sample of Polish adolescents

**DOI:** 10.1038/s41598-026-49533-9

**Published:** 2026-05-04

**Authors:** Paweł Strojny, Aleksandra Zajas, Patrycja Kiszka, Sylwia Starzec, Zsolt Demetrovics, Orsolya Király

**Affiliations:** 1 Institute of Applied Psychology, Faculty of Management and Social Communication, Jagiellonian University, Stanisława Łojasiewicza 4, Kraków, 30-348 Poland; 2 Doctoral School in the Social Sciences, Jagiellonian University, Kraków, Poland; 3 College of Education, Psychology and Social Work, Flinders University Institute for Mental Health and Wellbeing, Flinders University, Bedford Park, Adelaide, SA Australia; 4 Institute of Psychology, ELTE Eötvös Loránd University, Budapest, Hungary; 5 Centre of Excellence in Responsible Gaming, University of Gibraltar, Gibraltar, Gibraltar

**Keywords:** Gaming disorder, Gaming motivation, Introjected regulation, Behavioral addictions, Games, Human behaviour, Addiction

## Abstract

This study provides prevalence estimates of gaming disorder (GD) risk among Polish adolescents and validates a Polish translation of the Gaming Motivation Inventory (GMI). Moreover, it explores the role of introjected regulation in GD using a comprehensive motivational model. A representative sample of 1060 adolescents (930 gamers) completed measures of GD symptoms, gaming motivation, and involvement. GD risk ranged from 7.7% to 10.8%, higher among boys (8.9%–12.2%) than girls (6.5%–7.5%). The Polish GMI demonstrated strong psychometric properties, supporting its use in assessing gaming motivations among Polish adolescents. Introjected regulation, escape and identity were among the motivations most strongly associated with GD symptoms, with introjected regulation emerging as the strongest predictor.

**Supplementary Information:**

The online version contains supplementary material available at 10.1038/s41598-026-49533-9.

## Introduction

As gaming becomes increasingly prevalent worldwide, gaming disorder (GD) has gained formal recognition—being included in the International Classification of Diseases by the World Health Organization and listed in the DSM-5 appendix (Emerging Measures and Models)^1,2^. In response to this recognition, the need for further research has grown^3–5^. It is crucial to understand both the prevalence of GD in various populations and the underlying factors that contribute to its development^6^. A multinational study conducted between 2011 and 2012 among 12,938 adolescents from seven European countries found that 1.6% met the full criteria for Internet Gaming Disorder (IGD), with an additional 5.1% considered at risk. Country-specific differences were also noted, with the highest prevalence observed in Greece (2.6%) and Poland (2.1%)^7^. More recent meta-analyses report pooled global GD prevalence among adolescents ranging from 3.1%^8^, 3.3%^9^, up to 4.6% ^10^. Noteworthy, the authors emphasize the high variability of the results obtained across studies, with prevalence estimates ranging from less than 1% (e.g.5%)^11^ to approximately 20.0% (e.g. 17.7%)^12^, depending on the methodologies employed. The rapid rise in gaming’s popularity, coupled with the constant emergence of new game types and genres designed to attract broader audiences, underscores the importance of continuously monitoring the prevalence of GD and examining the risks associated with these evolving trends.

To better understand the phenomenon of gaming, it is essential to examine the motivations driving individuals to play. Since gaming is primarily a voluntary activity that typically lacks tangible rewards, the underlying motivations are often complex and multifaceted. Several theoretical and measurement frameworks have been developed to capture this complexity. A robust framework for categorizing this complexity is Self-Determination Theory (SDT), which provides a motivational taxonomy based on the degree to which behavior is self-determined^13^. While gaming is commonly driven by intrinsic motivation—playing for the inherent enjoyment and satisfaction^14^— SDT suggests that risk arises when motivation shifts toward controlled forms of extrinsic regulation. In parallel, gaming-specific models have focused more directly on the content of players’ motives. For example, Yee’s^15^ model organized gaming motives into the broad domains of achievement, social, and immersion, whereas Demetrovics et al.^16^ identified a more differentiated set of motives including socialization, competition, escape, coping, fantasy, skill development, and recreation. More recently, Király et al.^17^ proposed the Gaming Motivation Inventory as a comprehensive framework integrating motives represented across earlier instruments while also extending them to reflect contemporary gaming contexts, including motives such as financial gain, coping, or destruction.

However, motivation can be influenced by situational factors and change over time within one individual^18^. New kinds of motives can also emerge as a response to technological and social changes^17^. These dynamics highlight the importance of ongoing research into the motivations for gaming. The Interaction of Person-Affect-Cognition-Execution (I-PACE) model provides a theoretical basis for this perspective, suggesting that GD develops through an interaction between predisposing ‘Person’ factors and situational triggers^19^. In this model, gaming motives function as core person-level characteristics that (together with other factors) influence how individuals engage with gaming stimuli.

Despite the growing body of evidence, the literature remains uneven in its coverage of specific motivational constructs. While escapism has been extensively examined across different measurement frameworks, other theoretically relevant motives have received far less systematic attention. Particularly relevant is introjected regulation, where gaming is driven by internal pressures, such as a need to bolster self-esteem or avoid feelings of guilt and shame. For instance, a meta-analysis by Bäcklund and colleagues^20^ found significant associations between GD symptoms and 23 of the 26 identified motivational factors. While motives related to emotional escape showed robust pooled correlations approaching *r* = .50, the strongest association overall was observed for introjected regulation (*r* = .68). Although this estimate was based on only three available studies and should therefore be interpreted with caution. Another meta-analysis supported these findings, identifying escapism as the strongest correlate of IGD (*r* = .40) among several motivations; however, introjected regulation was not included in that synthesis.^21^. Considering that multiple motives have shown meaningful associations with GD symptoms across studies, a comprehensive examination of gaming motivations is justified, while giving particular attention to those with the strongest prior empirical support.

Interestingly, the meta-analysis by Wang & Cheng also found that escapism had a greater impact in individualistic cultures compared to collectivistic ones, suggesting a cultural component in how motivations relate to GD^21^. Beyond the cultural context, motivations for gaming can be influenced by factors such as gender, age, and socioeconomic status^15–17,22,23^. Gender, in particular, plays an important role in shaping these motivations and their subsequent impacts. For example, a study by Laconi et al.^24^ revealed that for women, GD symptoms were predicted by specific gaming motives such as escape and competition, compared to male gamers, whose GD symptoms predictors were escape and coping. A study conducted upon adolescent gamers showed that for men escapism was a significant moderator of the relationship between gaming time and GD symptoms, whereas for women it was not^25^. The moderating role of gaming motivations is particularly noteworthy, as it can exacerbate the negative impacts of other factors^25,26^. In general, differences in gaming motivations between men and women are influenced by a combination of societal, cultural, and in-game factors that uniquely shape the gaming experiences and preferences of each gender^27^.

As gaming motivations became a focal point of discussion in the psychology of gaming, numerous questionnaires have been developed to assess them^14,16,28–30^. However, these tools have faced limitations due to inconsistencies in the motives they assess, making it challenging to compare results across different studies^20^. Additionally, the lack of uniformity among these tools has hindered a comprehensive examination of the motives for gaming, as most of them only cover a limited number of motives. In contrast, the Gaming Motivation Inventory (GMI)^17^ offers a multidimensional approach to assessing gaming motivation. This tool tackles the challenge of comparing study results from different questionnaires by incorporating a wide range of motives identified in previous tools, while also introducing new motivates that reflect the contemporary realities of players. Despite being a recent development, the GMI is gaining popularity in the scientific community, demonstrating its value through its application in research^25,26,31–36^.

The present study had three main objectives. First, and most importantly, to present new findings on the prevalence of GD risk in the Polish adolescent population based on a representative sample. Second, to validate the GMI to Polish language. Third, to investigate the associations between gaming motivations and GD symptoms .

## Methods

### Participants and procedure

Respondents were children of individuals registered in ‘Ariadna’, a nationally representative research panel in Poland. The study was conducted in March 2023 using the Computer-Assisted Web Interviewing (CAWI) method. The sample was collected using a random quota sampling method from a panel that reflects the structure of the Polish adolescent population aged 13–17 with respect to age, parents’ education, parents’ employment status, and size of place of residence. Participation in the study was not available to all panel members but limited to those randomly selected. Parents received the survey link and passed it on to their child. Therefore, informed consent was obtained from legal guardians of all participants. Parents also completed a separate set of questions unrelated to the present study, as the data were originally collected as part of a broader project focusing on natural language processing^37^. The language data collected from parents has also been published in Strojny et al., 2024^38^. Parents were instructed to avoid interaction while adolescents completed the questionnaire; however, parental presence was not formally assessed. Participation was voluntary, and respondents received loyalty points in the ‘Ariadna’ system (awarded to the parent) as compensation. These points are exchangeable for various material rewards from a dedicated prize catalog, which served as the primary incentive for participation. The sample consisted of 1060 participants aged 13–17. Among them, *N* = 930 participants (411 girls, 518 boys, 1 other; *M*_*age*_ = 14.46, *SD* = 1.39) responded ‘yes’ to the question ‘Have you ever played video games?’ and were included in the analyses.

The study received approval from the Ethics Committee at the Institute of Applied Psychology of the Jagiellonian University and conformed to the principles outlined in the Declaration of Helsinki.

All the methods employed are listed below. In line with the best practices, the data, materials, and procedure for the study are available for download at the Open Science Framework^37^. Statistical analyses were conducted using IBM SPSS Statistics for Windows (version 29.0; IBM Corp., Armonk, NY, USA; https://www.ibm.com/products/spss-statistics) and IBM SPSS Amos (version 29.0; IBM Corp., Armonk, NY, USA; https://www.ibm.com/products/structural-equation-modeling-sem). The study was not preregistered.

### Measures

The Polish translation of the 88-item Gaming Motivation Inventory (GMI) has been used to assess gaming motives (see Appendix A). The GMI is a comprehensive motivation inventory applicable to any gaming genre, designed to capture a broad range of motivational factors. It comprises 88 items adapted from previous tools for assessing gaming motives^17^. Its psychometric properties have been evaluated in a large sample of Hungarian-speaking gamers. The original scale includes 26 factors (e.g., Escape, Identity, Status, Introjected Regulation), corresponding to theoretically formulated motives, confirmed through factor analysis. The 26 motives are clustered into 6 higher-order motivational dimensions in the original framework (Mastery, Immersion/Escapism, Competition, Stimulation, Social, Habit/Boredom); however, the present analyses were conducted solely at the level of the 26 motive subscales. The intensity of any given motive was assessed through a seven-point Likert scale, ranging from ‘Definitely not applicable’ to ‘Definitely applicable’. The number of items that constitute a given motive ranges from 3 to 5. The mean score of any given motive ranges from 1 to 7, with higher scores indicating greater intensity of a given motive. The GMI was developed by an international team, with item wording originally drafted in English and translated into Hungarian for the original validation. For the present study, we translated the Polish version from the English item set supplied by the original authors using the back-translation procedure. Two psychologists (AZ & SS) conducted the translation independently. Their versions were then compared, and a unified version was agreed upon. The back-translation was performed by two additional psychologists blind to the original wording (PS & PK) and submitted to the original authors for evaluation (OK & ZD). All translators had documented upper-intermediate (B2 or higher) English proficiency. The final consensus was reached by discussing significant differences in the back translation compared to the original English version.

Gaming involvement was assessed with the Gaming Involvement Scale (GIS)^36^. Participants were asked how much time they spent on six game-related activities (gaming, watching game-related videos, thinking about video games, reading game-related content, talking to other people about video games, and considering game-related purchases) during an average working day and an average weekend day (the values provided by the subjects were multiplied by 5 and 2, respectively, and then added to obtain weekly values). Gaming itself was treated as a separate metric of direct gaming involvement while the other five constitute the metric of indirect gaming involvement.

The severity of gaming disorder symptoms was assessed with two screening tools: Gaming Disorder Test (GDT)^39,40^ and Internet Gaming Disorder Scale–Short-Form(IGDS9-SF)^41,42^. The GDT comprises four items aligned with the World Health Organization’s definition of gaming disorder^40^. Participants responded on a five-item Likert scale. The higher the average, the greater the intensity of gaming disorder symptoms. A participant who answers ‘4’ or ‘5’ to each of the four questions can be considered as meeting the screening criteria. The IGDS9-SF includes nine items corresponding to the conceptualization of the Internet Gaming Disorder provided by American Psychiatric Association^41^. The participants respond on a five-item Likert scale. The higher the average, the greater the intensity of the symptoms of the gaming disorder. The established cutoff for differentiating disordered gamers from non-disordered gamers is 36 ^41^.

### Statistical analysis plan

First, we estimated the prevalence of GD risk with the two different methods stemming from the two diagnostic frameworks^1,2^. As not all respondents were gamers and we expected that the percentage of gamers might differ by gender, we reported the percentage of individuals at risk of GD separately for the full sample (corresponding to the adolescent population) and the gamer subsample.

We evaluated the internal consistency of each of the 26 factors using Cronbach’s alpha. We compared the values with those of the original study, which ranged between α = 0.75 and α = 0.93. Then we conducted confirmatory factor analysis (CFA) on each of the 26 factors separately using Amos software. For factors consisting of three items, the degrees of freedom in their structural models equaled 0, making model fit indices noninformative. Therefore, only factor loadings were informative within these models. We assumed a factor loading > 0.60 to be acceptable, following the authors of the original version. For factors with four or more items, we calculated several fit indices, including the Comparative Fit Index (CFI), the Root Mean Square Error of Approximation (RMSEA), and Standardized Root Mean Square Residual (SRMR). We applied the following thresholds to assess the goodness of fit of the models. CFI ≥ 0.95; RMSEA < 0.08, SRMR ≤ 0.10^43^.

Finally, we calculated the average scores for each motivation for the whole sample and gender subsamples. Gender differences of the intensity of gaming motivations were examined using *t*-test for independent samples. In several cases, the equal variances criteria were not met, and Welch’s correction was reported instead. Relationships between gaming motivations and gaming involvement were measured using Spearman’s correlation due to the high skewness of their distributions. In order to assess the relationships between individual motivations and the severity of gaming disorder symptoms (measured with GDT and IGDS9-SF), Pearson’s correlations were performed. Then, we conducted two separate linear regression analyses (one for each GD severity measure) using the motivations which were strongly (*r* > .50) correlated with GD severity as predictors.

To evaluate gender differences, we employed both independent sample *t-*tests, as well as multigroup CFAs to test for factor loading differences between groups. If Levene’s test showed unequal variances in groups, Welch’s correction was reported instead of the *t* statistic. In addition, to test whether the associations between gaming motives and disordered gaming symptoms varied by gender, we conducted a series of moderation analyses using Model 1 of the PROCESS macro. Each GMI motive was tested separately as a predictor, gender as the moderator, and GD symptom severity, measured with the GDT and the IGDS9-SF, as the outcome.

## Results

### Descriptive statistics

Participants were recruited as the children of adults enrolled in a nationally representative panel of Polish citizens, selected to reflect variation in education, urbanization, and employment status. Parents’ ages ranged from 29 to 73 years (*M* = 41.47, *SD* = 7.71). The average number of completed years of education among parents was 15 years (*SD* = 3.77). A total of 839 teenage participants (79.0%) were raised in two-parent households, closely aligning with the national statistic of 75.4%^44^. On average, there were 3.81 individuals in the household including the participant (*SD* = 1.09); this statistic for the population is 3.7% considering only households including at least one child^45^. Among the 1,060 parents, 964 (91%) were economically active. 854 worked full-time, 110 worked part-time or casually, 96 were not economically active, including 22 who were registered as unemployed. These figures correspond with Poland’s estimated percentage of economically active citizens (which is 90.6% for the dominant 40–44 age bracket)^46^. Regarding place of residence, 21.7% of the sample lived in rural areas, 11.5% in cities with up to 20,000 residents, 24.2% in cities with up to 100,000, 13.3% in cities with up to 200,000 and 29.2% in cities with more than 200,000 residents. These figures suggest a slight underrepresentation of residents of rural areas (40.5% of Polish residents)^47^. Of the sample of 1,060 adolescents surveyed, 128 were excluded for not playing video games. An additional 2 observations were excluded due to missing data.

### Prevalence of GD risk

We used two alternative methods to estimate the prevalence of GD risk. According to the recommendations for the use of the GDT, individuals who responded with a ‘4’ or ‘5’ to all items were classified as at risk for GD ^40^. As a result, prevalence rates for GD risk (ICD-11) were 9.4% in the total sample and 10.8% (*n* = 100) among gamers. According to the recommendations for the IGDS9-SF, individuals with a total score of 36 or higher are considered at risk for Internet GD ^41^. Using this criterion, 82 participants met the DSM-5 criteria for IGD, resulting in a prevalence rate of 7.7% in the overall sample and 8.8% among gamers. Gender-specific prevalence rates are presented in Table 1.

**Table 1:** Prevalence of gaming, Gaming Disorder risk (ICD-11), and Internet Gaming Disorder risk (DSM-5) in the total sample and by gender.

	Boys (*n* = 562)		Girls (*n* = 495)	Non-binary (*n* = 1)	Total (*N* = 1060)
Gamers	518 (92.2%^a^)		411 (83.0%^a^)	1 (0.1% ^a.b^)	930 (87.7% ^b^)
Non-gamers	44 (7.8%^a^)		84 (17.0%^a^)	0	128 (12.1% ^a^)
GD risk (ICD-11)	63 (12.2% ^c^)		37 (0.0% ^c^)	0	100 (10.8% ^c^)
IGD risk (DSM-5)	50 (9.7% ^c^)		32 (7.8% ^c^)	0	82 (8.8% ^c^)

^a^ Percentage relative to the gender subgroup (e.g., the proportion of all boys who are gamers). ^b^ Percentage relative to the total sample (*N* = 1060). ^c^ Prevalence rate among gamers (calculated relative to the number of gamers within that specific subgroup). GD = Gaming disorder as defined in the International Classification of Diseases by the World Health Organization^2^. IGD = Internet Gaming Disorder as defined in the Diagnostic and Statistical Manual of Mental Disorders by the American Psychiatric Association^1^. Gamers = Participants who responded ‘yes’ to the question ‘Have you ever played video games?’.

The GD risk was also assessed in terms of its criteria represented in four GDT items specifically among the group of gamers (*n* = 930). We counted the proportion of respondents who met each criterion by responding “Often” or “Very often” to a given GDT item. First three criteria, concerning difficulty with controlling the gaming activity, giving increasing priority to gaming and continuing despite negative consequences, were met by 25.1% − 27.7% of gamers. The last criterion, which was experiencing significant problems due to gaming behavior, was relatively less frequent and was met by 18.3%of this group. The results broken down by gender are provided in Table 2.

**Table 2:** Prevalence of Gaming Disorder criteria (ICD-11) among gamers by gender.

Having difficulty controlling one’s gaming activity	Boys (*n* = 518)	Girls (*n* = 411)	Non-binary (*n* = 1)	Total (*n* = 930)
	154 (29.7%)	83 (20.2%)	0	237 (25.5%)
Giving increasing priority to gaming over other life interests and daily activities	168 (32.4%)	90 (21.9%)	0	258 (27.7%)
Continuing gaming despite the occurrence of negative consequences	144 (27.8%)	89 (21.7%)	0	233 (25.1%)
Experiencing significant problems in life (e.g., personal, family, social, education, occupational) due to the severity of one’s gaming behavior	101 (19.5%)	69 (16.8%)	0	170 (18.3%)

A criterion was classified as “met” if the participant responded “Often” (4) or “Very often” (5) on the 5-point Likert scale for the corresponding GDT item.

### The structure of the GMI

Before examining motive–GD associations, we first evaluated the psychometric properties of the Polish GMI to ensure that the motivation scales functioned adequately in the present sample. Each GMI subscale demonstrated very good or excellent reliability (Table S6), with Cronbach’s alpha values ranging from 0.84 (Amotivation) to 0.94 (Game Skills, Finance, and Escape).

For subscales comprising only three items, it was not possible to estimate goodness-of-fit indices; therefore, only factor loadings were reported. In all other cases the SRMR and CFI indexes indicated an excellent fit, the RMSEA in some cases was higher than expected. However, it has been demonstrated that RMSEA can wrongly indicate a poor fit in simple models with few degrees of freedom 48. Factor loadings ranged between 0.65 and 0.93. Correlations among the 26 motives ranged from *r* = .01 (*p* = .790; for Recreation and Financial motives) to *r* = .91 (*p* < .001; for Game skills and Exploration & Mechanics motives) and can be seen in Supplemental Table S2 in the online Supplementary Information.

Gender-based differences in factor loadings of the GMI were found to be small. Only two of the eighty-eight items showed differences in loadings of 0.10 or more. A detailed comparison can be found in Table S3 in the Supplementary Information.

### Associations of motives, gaming engagement, and gaming disorder symptoms

The average scores for each motive ranged from 3.01 (Financial) to 5.42 (Recreation). Boys scored significantly higher than girls on all motives except for Amotivation, Boredom, Financial, where there were no significant differences between the two genders. The effect sizes were small or moderate (Cohen’s d values varied between 0.18 and 0.45). Full descriptive statistics for each motive are provided in Table S1 in the Supplementary Information.

Spearman’s correlation revealed that twenty-four motives were significantly positively correlated with both direct and indirect gaming involvement. The strength of these correlations was low to moderate (max *rho* = 0.36). Financial motive was not correlated with either direct or indirect gaming involvement, while Amotivation showed a weak negative correlation only with direct gaming involvement. The results are shown in Table S4 in Supplementary Information.

All gaming motives were positively correlated with both measures of problematic gaming, the GDT and the IGDS9-SF. For gaming disorder symptoms assessed by the GDT 40, three correlation coefficients reached or exceeded the conventional threshold of *r* = .50, indicating a strong correlation: Identity, Introjected Regulation, and Status. For Internet Gaming Disorder symptoms assessed by the IGDS9-SF ^41^, there were eight strong correlations: Completion, Coping, Escapism, Fantasy, Destruction, and again Identity, Introjected Regulation, and Status. The results are shown in Table S5 in Supplementary Information.

Subsequently, we conducted two linear regression analyses to test the motives strongly correlated with GD symptoms and IGD symptoms as their predictors. Assumptions for linear regression were tested prior to analysis. The Durbin-Watson statistic of 1.90 in the model predicting GD and 1.95 in the model predicting IGD indicated a minor, but not problematic autocorrelation among the residuals. Multicollinearity was assessed using the Variance Inflation Factor (VIF), with values for predictors ranging from 3.82 to 6.09 in the model predicting GD symptoms and from 2.50 to 7.28 in the model predicting IGD symptoms, suggesting moderate but acceptable multicollinearity. The Breusch-Pagan test for heteroscedasticity yielded a significant test statistic of 39.90 (*p* < .001) for GD symptoms and 78.81 (*p* < .001) for IGD symptoms, indicating some heteroscedasticity in the data. Therefore, robust standard errors were applied to address this issue. Results are presented in Tables 3 and 4.

**Table 3:** Linear regression analysis of Gaming Disorder symptoms predicted by selected gaming motives – parameter estimates with robust standard errors.

					95% CI	
	B	Robust SE	t	*p*	LLCI	ULCI
F(3;926) = 183.89; *p* < .001; R²adj. = 0.37						
(Constant)	4.28	0.32	13.34	< 0.001	3.65	4.91
Identity	− 0.20	0.22	− 0.88	0.382	− 0.64	0.24
Introjected Regulation	1.49	0.15	9.76	< 0.001	1.19	1.79
Status	0.24	0.19	1.29	0.199	− 0.13	0.60

Note. Dependent variable: IGD symptoms. *B* = regression coefficient; *Robust SE* = Robust standard error; *t* = test statistic; *p* = significance; *LLCI* = lower-level confidence interval; *ULCI* = upper-level confidence interval.

**Table 4:** Linear regression analysis of Internet Gaming Disorder symptoms predicted by selected gaming motives – parameter estimates with robust standard errors.

					95% CI	
	B	Robust SE	t	*p*	LLCI	ULCI
*F*(8;921) = 101.94; *p* < .001; *R*²adj. = 0.47						
(Constant)	5.79	0.65	8.87	< 0.001	4.51	7.07
Completion	0.73	0.07	3.23	0.001	0.29	1.17
Coping	0.12	0.11	0.34	0.736	− 0.57	0.80
Escapism	1.24	0.07	3.94	< 0.001	0.62	1.85
Fantasy	− 0.55	0.08	-1.57	0.117	-1.23	0.14
Destruction	0.72	0.07	3.40	0.001	0.30	1.13
Identity	− 0.85	0.08	-1.80	0.071	-1.78	0.07
Introjected Regulation	2.85	0.09	10.24	< 0.001	2.31	3.40
Status	− 0.14	0.10	− 0.39	0.695	− 0.82	0.54

Note. Dependent variable: IGD symptoms. *B* = regression coefficient; *Robust SE* = Robust standard error; *t* = test statistic; *p* = significance; *LLCI* = lower-level confidence interval; *ULCI* = upper-level confidence interval.

The results of the first linear regression analysis indicate that the model significantly predicted GD symptoms and explained approximately 37% of the variance. Among the predictors, only Introjected Regulation showed a significant and positive relationship with the dependent variable. Identity and Status were not significant predictors in this model.

The second linear regression analysis revealed that the model significantly predicted Internet Gaming Disorder symptoms, explaining about 47% of the variance. Significant predictors included Introjected Regulation, Escapism, Completion and Destruction. Other predictors, such as Coping, Fantasy, Identity and Status, were not statistically significant in predicting IGD symptoms.

Finally, we conducted a series of moderation analyses using Model 1 of the PROCESS macro 49 with gender as the moderator, all GMI motives as independent variables, and both GD and IGD as dependent variables. Only one interaction was statistically significant and it was Financial motivation. Specifically, Financial motivation significantly interacted with gender in predicting both GD (*F*(3, 925) = 58.08, *R*² = 0.16, *p* < .001] and IGD [*F*(3, 925) = 74.49, *R*² = 0.19, *p* < .001], such that higher Financial motivation was associated with increased symptom severity in both boys and girls, but this association was stronger among boys than girls (GDT interaction: *b* = − 0.42, *t*(925) = − 2.94, *p* = .003; IGD interaction: *b* = − 0.98, *t*(925) = − 3.28, *p* = .001). The conditional effects of financial motivation on GDT were *b* = 1.09 (*p* < .001) for boys and *b* = 0.67 (*p* < .001) for girls; for IGD, *b* = 2.59 (*p* < .001) for boys and *b* = 1.62 (*p* < .001) for girls. Given space limitations and the largely null pattern for other motives, we report only this robust interaction in the main text.

## Discussion

### The prevalence of gaming disorder risk

The first aim of the present study was to provide information regarding the prevalence estimate of GD risk in the population of Polish adolescents. We obtained a relatively high prevalence estimate of GD risk in the entire sample (including non-gaming participants), ranging from 7.7% to 9.4%, depending on the measurement method. When considering only individuals who had played games at least once in their lives, the estimated prevalence ranged from 8.8% to 10.8%. The prevalences obtained are relatively high, but within the limits obtained previously 9, suggesting both their reliability and the concerning scale of the issue—approximately 1 in 10 Polish adolescents may be at risk of developing GD. Gender played a role, with men being more likely to suffer from GD than girls, both in the overall population and within the gamer subsample.

The only previous research on GD risk prevalence among Polish adolescents was conducted in early 2012, more than a decade before the current study. It was also carried out using different methods, as the diagnostic tools currently used were developed following the formal recognition of GD by the APA 1 and the WHO 2. Results obtained then and now should be compared having these in mind. According to Müller and colleagues 7, 60.5% of the entire sample of adolescents from seven European countries played games. There was a significant gender difference with 84.7% of boys and 42.8% of girls gaming. In our current sample, this figure was considerably higher, with 87.7% of adolescents reporting gaming, and the gender gap was markedly narrower: 92% of boys and 83% of girls reported gaming. This demonstrates the increasing popularity of gaming as a form of entertainment and the growing appeal of games designed for girls. It also shows the progressive equalization of both accessibility and cultural acceptance of girls gaming. Obviously, this trend could result in a tendency to increase in the proportion of both boys and girls at risk of GD.

According to the results of Müller and colleagues, 5.1% of the entire sample were at risk of GD while 1.6% met full criteria. Only data regarding participants fully meeting GD criteria are available for the Polish subsample, 2.0% of surveyed Poles (3.6% of boys and 0.6% of girls) exhibited GD risk a decade ago 7. Given that in our study we used screening tools (i.e., designed to identify people at risk), which do not have specific thresholds for distinguishing people at risk from those who meet full GD criteria, we used the percentage of people at risk taken from the previous study as a benchmark. However, even in this case, the percentage of such individuals in the current study is higher, ranging between 7.7% (IGD) and 9.4% (GD). At the same time, gender differences have largely blurred, although we recorded a higher percentage of those at risk for GD among boys (8.9% and 11.2%, respectively) compared to girls (6.5% and 7.5%, respectively). This proportion of adolescents potentially affected by GD is alarming and and highlights the need for more frequent monitoring in the future.

### Psychometric characteristics of the Polish version of the GMI

The second aim of our study was to explore the psychometric characteristics of GMI in the sample of Polish-speaking adolescents. The study was carried out on a sample of adolescents who came from families representative of the Polish population. The mean age of the sample was 14 ± 1 years. The gender ratio was close to balanced, with 55.5% of the sample being male. Although the original GMI validation involved mainly adults with the majority of men (89.3%) ^17^, the present study contributes valuable insight into the gaming motivations of a younger, more gender-balanced demographic.

Our results confirmed a good fit and excellent internal consistency of motivational factors. The internal consistency was found to be very high, only in a few cases dropping to an alpha level below 0.9. The CFA results were similar or higher to those obtained by the original authors.

The mean scores for individual motivations ranged from 3.01 (Financial) to 5.42 (Recreation). These findings align with the original study, where Recreation was also the most highly endorsed motive (*M* = 6.00), and Financial was the least (*M* = 1.33). Interestingly, respondents in our study reported more than twice the importance of the financial motive first identified by Király et al. ^17^. While this may reflect generational shifts in the perceived value of gaming as a potential source of income, we suggest that the age of our sample is a key factor. Adolescents may view gaming as a legitimate path to financial gain—either presently or in the near future—whereas adults may have already committed to alternative career paths.

To test the construct validity of the motivational dimensions in the model, we checked their associations with gaming engagement, GD symptoms, and gender. Gender differences revealed that most motivations were more strongly endorsed by boys than girls. Since almost all of the effect sizes were small, there is no reason to conclude that differences in motives are strictly related to gender. These findings align with prior research 50, suggesting that the intensity of gaming motives is associated with other factors such as psychological context, game characteristics, or personality.

Low but statistically significant correlations between individual motives and gaming involvement (both direct and indirect) confirm the validity of the tool. In this context, the very low correlation coefficients for Amotivation, Boredom, and Financial motives with gaming involvement may seem surprising. However, the correlations between these motivations and risk of disordered gaming turned out to be moderate. Taken together, these results suggest that these particular motives may be especially important for distinguishing individuals in the later stages of disordered gaming development, characterized by a shift from playing for gratification toward playing for compensation 19,51. However, this issue requires further longitudinal studies. As predicted, there were strong correlations between motives that were previously associated with GD risk: Completion, Coping, Escapism, Fantasy, Destruction, Identity, Introjected Regulation, Status. These results further reinforce the relevance of the GMI for assessing motivational patterns associated with gaming-related dysfunction.

### The role of the gaming motivations

The exploratory analyses revealed a broadly positive pattern of associations between gaming motivations and GD symptoms, suggesting that problematic gaming is linked not to a single dominant motive but to a wider motivational configuration. Although several motives showed particularly strong correlations (Completion, Coping, Destruction, Escapism, Fantasy, Identity, Introjected Regulation, Status), the overall pattern indicates that GD symptoms may emerge from overlapping motivational processes rather than from one isolated pathway. Several of the strongest associations observed in the present study align with previously identified motivational pathways linked to GD. Coping- and escapism-related motives may reflect the use of gaming as an emotion-regulation strategy, whereas completion- and achievement-oriented motives may relate to persistence and goal pursuit within structured game systems. Identity- and status-related motives suggest that social evaluation and self-concept processes may also contribute to the maintenance of intensive gaming patterns. The presence of strong correlations across conceptually distinct domains supports the view that GD symptoms are embedded in a multifaceted motivational landscape rather than driven by a single factor. Among all examined motivations, introjected regulation uniquely emerged as a significant predictor in both GD and IGD regression models; with Beta coefficients considerably higher than those of other significant predictors in both models (including Escapism motivation which is better covered by empirical research). However, this finding should be interpreted within the broader exploratory context of the present analyses.

This result aligns with prior research: a meta-analysis 20, identified three studies employing tools capable of measuring Introjected Regulation, which reported strong associations between this motivation and GD symptoms 52–54. Taking into that Introjected Regulation reflects an internalized feeling of necessity, pressure, or compulsion to play in order to feel good about oneself. The subscale includes three items directly referring to the subjective sense of necessity: specifically, to ‘feel good about oneself,’ ‘to avoid feeling bad about oneself,’ and simply the feeling of ‘needing’ to play with no specific reason. However, the large effect size and consistent occurrence of this motivation in both models including IGD and GD suggest that its more detailed understanding may be essential to comprehending the mechanisms governing the dynamics of GD.

Based on the results of cross-sectional studies, such as the current study, it is impossible to determine the direction of a possible cause-effect relationship between GD symptoms and this motivation. Perhaps, in accordance with the postulates of the Interaction of Person-Affect-Cognition-Execution (I-PACE) model 19, which places particular emphasis on the phenomenon of gradual replacement of gaming for gratification with compulsive gaming, high scores on the Introjected Regulation scale are an emanation of the process of switching to the compulsive side observed by the subjects with disorders (Introjected Regulation motivation as a result of GD). On the other hand, it is also possible that the subjects who do not yet meet the behavioral criteria of GD (e.g. specific behaviors, harm, cheating on loved ones or withdrawal syndrome) are able to see its early symptoms in themselves in the form of relatively high levels of this ‘compulsion’ to play (Introjected Regulation as the cause of GD). Moreover, taking into account that self-report tools clearly dominate in GD research, there is also a third possibility. Namely, some other variable or their constellation predetermines some of the subjects to be particularly sensitive to perceiving in themselves both the ‘compulsion’ reflected in the items of this subscale and the critical assessment of their functioning reflected by high scores on GD screening tools. Longitudinal studies are necessary to establish the answer, and it would be particularly useful to support self-report methods with a clinical diagnosis of GD.

## Study limitations

The primary limitation of the present study is the cross-sectional design, which restricts our ability to draw conclusions about causal relationships or differences in motive intensity depending on the stage of GD development process. Questions about well-being, mental health, preferred game genres, and individual differences were not included in our survey, limiting the possibilities of controlling their impact. Although we successfully obtained a representative sample of Polish adolescents, a notable limitation is the exclusive use of online data collection, which inherently excluded individuals without Internet access. However, according to the 2023 report by the Polish Central Statistical Office, 99.8% of households with children had Internet access. Therefore, even assuming that children from the remaining 0.2%—who are unlikely to be at risk of GD for obvious reasons—were excluded, their absence would not have a meaningful impact on the results obtained 55. Additionally, parents could be present during questionnaire completion but were explicitly instructed to refrain from communication with the adolescent; however, parental non-involvement was not formally verified, and thus should be interpreted as a procedural instruction rather than a confirmed condition.

Another limitation of the study is the fact that we used new tools that are not fully comparable to the tool used by Müller. In the future, it would also be advisable to estimate the prevalence based on clinical tools that provide information about actual diagnosis of GD. A potential limitation of our study is the use of the question: ‘Have you ever played computer games?’ to distinguish between those who have played at least once and those who have never played. However, this question does not differentiate between individuals who play regularly and those who have played only once and then stopped. We addressed this by considering the declared level of direct gaming involvement. Among those who answered affirmatively to the control question, 62 participants reported not spending any time playing games during the week. Interestingly, within this group, the lowest indirect gaming involvement was a minimum of 150 min per week, suggesting that even adolescents who do not play regularly still dedicate a significant amount of time to activities such as thinking about gaming or watching gaming-related videos.

Regarding the validation of the Polish translation of GMI, it should be noted that although the GMI was created by an international team and items were originally drafted in English before being translated into Hungarian for the original validation, the full English version of the inventory has not yet been psychometrically validated in an English-speaking sample. Accordingly, the Polish version was translated from an English item set that has not been validated as a complete inventory. To minimize potential bias at the adaptation stage, we used an independent forward–backward translation procedure with back-translators blinded to the original wording, and the back-translation was reviewed in collaboration with the original GMI authors.

Additionally, we obtained relatively high correlations between some of the individual motives, which may indicate a lack of independence between certain factors, potentially influenced by the younger age of our sample. While the original GMI was validated primarily with adult participants, adolescents may have more difficulty distinguishing between nuanced motivational dimensions. However, we decided not to attempt merging the correlated subscales. Nevertheless, the modified GMI should be regarded rather as a comprehensive collection of the 26 most important gaming motives from which the researchers can select those of interest. As such, it should not be considered a tool for distinguishing between specific motives—at least not within adolescent samples.

## Conclusions

In this study, we aimed to provide an up-to-date prevalence estimate of GD risk among Polish adolescents and to adapt a comprehensive and applicable inventory of gaming motivations for all genres. In summary, we noticed higher percentage of adolescents at risk of GD compared to data from a decade ago, particularly among girls. Although the study was not longitudinal, and the conceptualization and operationalization of GD differed between our study and the previous one, the findings may suggest a rising trend in the proportion of adolescents at risk of GD over time. We also found that specific gaming motivations were significantly associated with both gaming involvement and the severity of GD symptoms. The Polish translation of the GMI has good psychometric properties and can be applied in situations where it is necessary to consider the motives of gamers. Particularly in research, education, and industrial contexts.

## Supplementary Information

Below is the link to the electronic supplementary material.

Supplementary Material 1

## Data Availability

Data collected and analyzed during this research can be found at: https://osf.io/ay296/.
