# Supplementary material for: Introjected regulation is the primary predictor of gaming disorder symptoms across WHO and APA criteria in a representative sample of Polish adolescents
Source: Sci Rep. 2026 May 4;16:23135. doi: 10.1038/s41598-026-49533-9 (PMC13396395; doi:10.1038/s41598-026-49533-9)
Supplement: Supplementary file 1 — Supplementary Material 1 [file 41598_2026_49533_MOESM1_ESM.docx]

**Supplementary Information**

Gaming Motivation Inventory – Polish version

Dlaczego grasz w gry wideo? Gram w gry wideo …

**Rozwój**

1. ponieważ lubię uczucie ciągłego awansowania.

2. ponieważ lubię awansować w grach.

3. ponieważ lubię, gdy przechodzę na kolejny poziom/etap w grach.

**Amotywacja**

4. kiedyś miałem/miałam dobre powody, ale teraz zadaję sobie pytanie, czy powinienem/powinnam dalej grać.

5. szczerze mówiąc, nie wiem, mam wrażenie, że marnuję czas.

6. to przestało być jasne, czasami zadaję sobie pytanie, czy to dla mnie dobre.

**Autonomia**

7. ponieważ sam/sama mogę ustalać co robię w grach.

8. ponieważ mogę grać w gry zgodnie z moimi preferencjami.

9. ponieważ dają mi ciekawe opcje i wybory.

10. ponieważ w grach doświadczam dużo swobody.

**Nuda**

11. ponieważ się nudzę.

12. dla zabicia czasu.

13. ponieważ nie mam nic innego do roboty.

**Kompetencja**

14. ponieważ gdy dobrze mi idzie w grze, to dobrze się czuję ze sobą.

15. ponieważ odnoszone przeze mnie sukcesy podnoszą mi samoocenę.

16. ponieważ czuję się bardzo kompetentny/kompetentna i skuteczny/skuteczna podczas gry.

**Rywalizacja**

17. ponieważ lubię rywalizować z innymi.

18. ponieważ lubię wygrywać.

19. ponieważ lubię być lepszy/lepsza od innych.

**Ukończenie**

20. dopóki nie skończę 100% gry, zbieram wszystko, co się da.

21. dopóki nie odblokuję wszystkich osiągnięć.

22. dopóki nie opanuję wszystkich elementów gry.

**Radzenie sobie**

23. ponieważ pomagają mi wyładować złość.

24. ponieważ pomagają mi się odstresować.

25. ponieważ wprawiają mnie w lepszy nastrój.

**Eskapizm**

26. aby uniknąć myślenia o realnych problemach i zmartwieniach.

27. ponieważ granie pomaga mi zapomnieć o codziennych kłopotach.

28. aby zapomnieć o niemiłych rzeczach lub urazach.

29. ponieważ granie pomaga mi uciec od rzeczywistości.

**Eksploracja + Mechanika**

30. ponieważ lubię odkrywać różne elementy lub możliwości gry.

31. ponieważ lubię eksperymentować z różnymi sposobami grania.

32. ponieważ lubię dochodzić do tego, jak działają poszczególne elementy gry.

33. ponieważ lubię dogłębnie odkrywać/uczyć się mechaniki gry.

**Fantastyka**

34. ponieważ mogę robić rzeczy, które nie są możliwe w rzeczywistości lub nie wolno mi ich robić.

35. ponieważ mogę być w innym świecie.

36. ponieważ mogę być przez chwilę kimś innym lub w innym miejscu.

37. ponieważ czuję się zanurzony/zanurzona w wirtualnym świecie.

**Finansowe**

38. ponieważ mam możliwość zarabiania pieniędzy.

39. ponieważ mam szansę na dodatkowy dochód.

40. ponieważ mogę zarobić trochę pieniędzy.

**Umiejętności w grze**

41. ponieważ lubię osiągać szczyt moich możliwości.

42. ponieważ lubię doskonalić określone umiejętności w grach.

43. ponieważ lubię ciągle udoskonalać swoją grę.

44. ponieważ lubię ćwiczyć i doskonalić grę.

**Tożsamość**

45. ponieważ granie w gry jest sensowną aktywnością.

46. ​​ponieważ gry są przedłużeniem mnie.

47. ponieważ są integralną częścią mojego życia.

48. ponieważ mają dla mnie osobiste znaczenie.

49. ponieważ gry są w harmonii z innymi zajęciami w moim życiu.

**Wewnętrzna regulacja**

50. ponieważ muszę grać, żeby czuć się dobrze ze sobą.

51. ponieważ inaczej czułbym/czułabym się źle ze sobą.

52. ponieważ czuję, że muszę grać regularnie.

**Rekreacja**

53. ponieważ gry są fajne.

54. dla zabawy.

55. żeby się zrelaksować.

**Rozwój umiejętności**

56. ponieważ granie wyostrza moje zmysły.

57. ponieważ poprawiają moje umiejętności.

58. ponieważ poprawiają moją koncentrację.

59. ponieważ poprawiają moją koordynację.

**Społeczność**

60. ponieważ mogę poznać nowych ludzi.

61. ponieważ lubię grać z innymi.

62. ponieważ czuję się związany/związana z innymi graczami.

63. ponieważ uważam relacje z innymi graczami za istotne.

**Pozycja społeczna**

64. dla prestiżu z bycia dobrym graczem.

65. ponieważ zdobywam uznanie i szacunek innych.

66. ponieważ inni postrzegają mnie jako kompetentnego gracza.

Jakie rodzaje gier preferujesz? Lubię gry wideo, które…

**Pobudzenie-działanie**

67. podnoszą poziom adrenaliny.

68. trzymają w napięciu.

69. podnoszą poziom ekscytacji.

70. są intensywne i dynamiczne.

**Współpraca**

71. pozwalają graczom na współpracę z innymi.

72. promują wspólną pracę jako grupa.

73. wymagają pracy zespołowej.

**Personalizacja**

74. pozwalają graczom personalizować obiekty w grze (np. awatar/pojazd/przedmioty…).

75. zapewniają graczom wiele opcji personalizacji.

76. pozwalają graczom na personalizację swoich przedmiotów/rzeczy/postaci tak, żeby dać poczucie wyjątkowości.

**Zniszczenie**

77. pozwalają graczom na robienie eksplozji.

78. wiążą się ze zniszczeniem.

79. pozwalają graczom coś popsuć.

**Grafika**

80. wizualnie zapierają dech w piersiach.

81. mają znakomitą grafikę.

82. mają dobrą grafikę, są po prostu piękne.

**Fabuła**

83. mają ciekawą fabułę.

84. mają rozbudowaną fabułę, która wzbudza moje emocje.

85. mają wciągającą narrację.

**Strategia**

86. wymagają strategicznego myślenia.

87. wymagają planowania z wyprzedzeniem i podejmowania strategicznych decyzji.

88. wymagają podejmowania decyzji taktycznych.

**Supplemental Table S1**

*Descriptive Statistics of Gaming Motives and Gender Differences in the Intensity of Gaming Motives*

|  | Total sample  (*n* = 930) | Girls  (*n* = 411) | Boys  (*n* = 518) |  |  |  |  |  | 95% *CI* | |  |
| --- | --- | --- | --- | --- | --- | --- | --- | --- | --- | --- | --- |
|  | *M (SD)* | *M* (*SD*) | *M* (*SD*) | Skewness | Kurtosis | *t* | *df* | *p* | *LL* | *UL* | Cohen’s *d* |
| Advancement | 5.15 (1.40) | 4.88 (1.51) | 5.37 (1.27) | -.80 | .42 | -5.22ᵃ | 799.27 | **<.001** | -.67 | -.30 | .35 |
| Amotivation | 3.82 (1.57) | 3.83 (1.60) | 3.81 (1.55) | -.12 | -.67 | .20 | 927.00 | .843 | -.18 | .22 | .01 |
| Autonomy | 5.13 (1.35) | 4.89 (1.44) | 5.32 (1.25) | -.76 | .47 | -4.85ᵃ | 814.28 | **<.001** | -.61 | -.26 | .33 |
| Boredom | 4.63 (1.51) | 4.66 (1.53) | 4.60 (1.49) | -.51 | -.26 | .58 | 927 | .563 | -.14 | .25 | .04 |
| Competence | 4.88 (1.48) | 4.59 (1.59) | 5.10 (1.36) | -.69 | .09 | -5.11ᵃ | 807.68 | **<.001** | -.70 | -.31 | .34 |
| Competition | 5.1 (1.44) | 4.8 (1.54) | 5.34 (1.30) | -.79 | .22 | -5.59ᵃ | 801.41 | **<.001** | -.72 | -.35 | .38 |
| Completion | 4.7 (1.57) | 4.4 (1.70) | 4.93 (1.42) | -.58 | -.24 | -5.03ᵃ | 796.05 | **<.001** | -.73 | -.32 | .34 |
| Coping | 4.7 (1.45) | 4.53 (1.50) | 4.83 (1.41) | -.53 | -.14 | -3.15 | 927 | **.002** | -.49 | -.11 | .21 |
| Escape | 4.4 (1.61) | 4.23 (1.68) | 4.52 (1.55) | -.43 | -.49 | -2.75 | 927 | **.006** | -.50 | -.08 | .18 |
| Exploration & Mechanics | 4.81 (1.44) | 4.56 (1.53) | 5.01 (1.33) | -.64 | .09 | -4.76ᵃ | 817.55 | **<.001** | -.64 | -.27 | .32 |
| Fantasy | 4.65 (1.52) | 4.44 (1.63) | 4.82 (1.41) | -.57 | -.17 | -3.70ᵃ | 813.62 | **<.001** | -.58 | -.18 | .25 |
| Financial | 3.01 (1.84) | 2.88 (1.83) | 3.10 (1.84) | .44 | -1.07 | -1.76 | 927 | .078 | -.45 | .02 | .12 |
| Game skills | 4.86 (1.48) | 4.57 (1.60) | 5.10 (1.34) | -.66 | .09 | -5.40ᵃ | 800.86 | **<.001** | -.72 | -.34 | .36 |
| Identity | 4.28 (1.53) | 3.99 (1.62) | 4.52 (1.41) | -.35 | -.44 | -5.20ᵃ | 815.52 | **<.001** | -.72 | -.33 | .35 |
| Introjected regulation | 3.91 (1.73) | 3.6 (1.79) | 4.15 (1.64) | -.17 | -.93 | -4.82ᵃ | 841.95 | **<.001** | -.77 | -.33 | .32 |
| Recreation | 5.42 (1.24) | 5.27 (1.31) | 5.53 (1.17) | -.76 | .40 | -3.16ᵃ | 829.44 | **.002** | -.42 | -.10 | .21 |
| Skill development | 4.68 (1.47) | 4.39 (1.56) | 4.92 (1.35) | -.58 | -.03 | -5.49ᵃ | 813.83 | **<.001** | -.72 | -.34 | .37 |
| Social | 4.68 (1.54) | 4.35 (1.6) | 4.94 (1.44) | -.52 | -.26 | -5.87ᵃ | 833.12 | **<.001** | -.79 | -.39 | .39 |
| Status | 4.39 (1.69) | 3.98 (1.78) | 4.72 (1.54) | -.46 | -.59 | -6.70ᵃ | 814.08 | **<.001** | -.96 | -.53 | .45 |
| Arousal-action | 4.93 (1.40) | 4.62 (1.46) | 5.18 (1.30) | -.63 | .16 | -6.13ᵃ | 829.84 | **<.001** | -.74 | -.38 | .41 |
| Cooperation | 4.78 (1.44) | 4.55 (1.54) | 4.96 (1.33) | -.64 | .20 | -4.24ᵃ | 809.97 | **<.001** | -.59 | -.22 | .28 |
| Customization | 4.99 (1.39) | 4.8 (1.47) | 5.13 (1.31) | -.57 | .09 | -3.62 | 927 | **<.001** | -.51 | -.15 | .24 |
| Destruction | 4.23 (1.57) | 3.84 (1.63) | 4.54 (1.46) | -.31 | -.56 | -6.75ᵃ | 832.49 | **<.001** | -.90 | -.49 | .45 |
| Graphics | 5.07 (1.37) | 4.88 (1.44) | 5.22 (1.30) | -.68 | .29 | -3.81ᵃ | 833.35 | **<.001** | -.53 | -.17 | .25 |
| Story | 5.06 (1.35) | 4.86 (1.40) | 5.21 (1.30) | -.64 | .17 | -3.90 | 927 | **<.001** | -.52 | -.17 | .26 |
| Strategy | 4.98 (1.35) | 4.74 (1.42) | 5.17 (1.26) | -.56 | .13 | -4.95 | 927 | **<.001** | -.61 | -.26 | .33 |

^a^ The result of Levene's test turned out to be statistically significant - the result was reported with Welch's correction.

**Supplemental Table S2**

*Zero-Order Correlations Between Gaming Motives*

|  | 1 | 2 | 3 | 4 | 5 | 6 | 7 | 8 | 9 | 10 | 11 | 12 | 13 | 14 | 15 | 16 | 17 | 18 | 19 | 20 | 21 | 22 | 23 | 24 | 25 |
| --- | --- | --- | --- | --- | --- | --- | --- | --- | --- | --- | --- | --- | --- | --- | --- | --- | --- | --- | --- | --- | --- | --- | --- | --- | --- |
| 1. Advancement | - |  |  |  |  |  |  |  |  |  |  |  |  |  |  |  |  |  |  |  |  |  |  |  |  |
| 1. Amotivation | .25^***^ | - |  |  |  |  |  |  |  |  |  |  |  |  |  |  |  |  |  |  |  |  |  |  |  |
| 1. Autonomy | .88^***^ | .24^***^ | - |  |  |  |  |  |  |  |  |  |  |  |  |  |  |  |  |  |  |  |  |  |  |
| 1. Boredom | .46^***^ | .42^***^ | .48^***^ | - |  |  |  |  |  |  |  |  |  |  |  |  |  |  |  |  |  |  |  |  |  |
| 1. Competence | .87^***^ | .31^***^ | .84^***^ | .49^***^ | - |  |  |  |  |  |  |  |  |  |  |  |  |  |  |  |  |  |  |  |  |
| 1. Competition | .89^***^ | .25^***^ | .83^***^ | .48^***^ | .85^***^ | - |  |  |  |  |  |  |  |  |  |  |  |  |  |  |  |  |  |  |  |
| 1. Completion | .79^***^ | .34^***^ | .76^***^ | .50^***^ | .80^***^ | .76^***^ | - |  |  |  |  |  |  |  |  |  |  |  |  |  |  |  |  |  |  |
| 1. Coping | .70^***^ | .30^***^ | .72^***^ | .55^***^ | .75^***^ | .68^***^ | .65^***^ | - |  |  |  |  |  |  |  |  |  |  |  |  |  |  |  |  |  |
| 1. Escape | .61^***^ | .41^***^ | .63^***^ | .55^***^ | .66^***^ | .59^***^ | .62^***^ | .84^***^ | - |  |  |  |  |  |  |  |  |  |  |  |  |  |  |  |  |
| 1. Exploration & Mechanics | .79^***^ | .26^***^ | .81^***^ | .45^***^ | .77^***^ | .75^***^ | .75^***^ | .74^***^ | .67^***^ | - |  |  |  |  |  |  |  |  |  |  |  |  |  |  |  |
| 1. Fantasy | .75^***^ | .32^***^ | .78^***^ | .48^***^ | .78^***^ | .71^***^ | .72^***^ | .83^***^ | .81^***^ | .81^***^ | - |  |  |  |  |  |  |  |  |  |  |  |  |  |  |
| 1. Financial | .22^***^ | .47^***^ | .21^***^ | .26^***^ | .30^***^ | .22^***^ | .34^***^ | .31^***^ | .42^***^ | .31^***^ | .34^***^ | - |  |  |  |  |  |  |  |  |  |  |  |  |  |
| 1. Game skills | .84^***^ | .25^***^ | .83^***^ | .45^***^ | .83^***^ | .80^***^ | .77^***^ | .77^***^ | .68^***^ | .91^***^ | .83^***^ | .31^***^ | - |  |  |  |  |  |  |  |  |  |  |  |  |
| 1. Identity | .69^***^ | .35^***^ | .67^***^ | .45^***^ | .76^***^ | .67^***^ | .70^***^ | .75^***^ | .74^***^ | .73^***^ | .79^***^ | .46^***^ | .74^***^ | - |  |  |  |  |  |  |  |  |  |  |  |
| 1. Introjected regulation | .54^***^ | .41^***^ | .51^***^ | .44^***^ | .63^***^ | .53^***^ | .64^***^ | .66^***^ | .71^***^ | .57^***^ | .68^***^ | .51^***^ | .59^***^ | .85^***^ | - |  |  |  |  |  |  |  |  |  |  |
| 1. Recreation | .71^***^ | .07^*^ | .75^***^ | .46^***^ | .66^***^ | .70^***^ | .56^***^ | .67^***^ | .51^***^ | .67^***^ | .64^***^ | .01 | .69^***^ | .55^***^ | .37^***^ | - |  |  |  |  |  |  |  |  |  |
| 1. Skill development | .76^***^ | .28^***^ | .75^***^ | .42^***^ | .77^***^ | .73^***^ | .70^***^ | .72^***^ | .66^***^ | .81^***^ | .78^***^ | .34^***^ | .83^***^ | .82^***^ | .65^***^ | .65^***^ | - |  |  |  |  |  |  |  |  |
| 1. Social | .71^***^ | .29^***^ | .70^***^ | .44^***^ | .74^***^ | .71^***^ | .67^***^ | .69^***^ | .63^***^ | .73^***^ | .71^***^ | .37^***^ | .75^***^ | .81^***^ | .71^***^ | .61^***^ | .79^***^ | - |  |  |  |  |  |  |  |
| 1. Status | .73^***^ | .32^***^ | .68^***^ | .43^***^ | .78^***^ | .72^***^ | .72^***^ | .70^***^ | .67^***^ | .74^***^ | .74^***^ | .44^***^ | .77^***^ | .88^***^ | .79^***^ | .53^***^ | .82^***^ | .84^***^ | - |  |  |  |  |  |  |
| 1. Arousal-action | .74^***^ | .23^***^ | .75^***^ | .42^***^ | .75^***^ | .72^***^ | .67^***^ | .71^***^ | .60^***^ | .75^***^ | .74^***^ | .25^***^ | .78^***^ | .69^***^ | .55^***^ | .70^***^ | .74^***^ | .70^***^ | .71^***^ | - |  |  |  |  |  |
| 1. Cooperation | .59^***^ | .26^***^ | .60^***^ | .37^***^ | .61^***^ | .59^***^ | .54^***^ | .54^***^ | .49^***^ | .62^***^ | .59^***^ | .29^***^ | .63^***^ | .61^***^ | .52^***^ | .51^***^ | .63^***^ | .78^***^ | .65^***^ | .71^***^ | - |  |  |  |  |
| 1. Customization | .70^***^ | .16^***^ | .73^***^ | .38^***^ | .67^***^ | .65^***^ | .59^***^ | .64^***^ | .55^***^ | .72^***^ | .69^***^ | .19^***^ | .73^***^ | .61^***^ | .46^***^ | .67^***^ | .67^***^ | .65^***^ | .61^***^ | .77^***^ | .68^***^ | - |  |  |  |
| 1. Destruction | .55^***^ | .31^***^ | .54^***^ | .41^***^ | .59^***^ | .53^***^ | .60^***^ | .60^***^ | .58^***^ | .60^***^ | .64^***^ | .44^***^ | .59^***^ | .69^***^ | .63^***^ | .40^***^ | .63^***^ | .64^***^ | .69^***^ | .67^***^ | .59^***^ | .57^***^ | - |  |  |
| 1. Graphics | .68^***^ | .16^***^ | .73^***^ | .40^***^ | .65^***^ | .65^***^ | .58^***^ | .61^***^ | .51^***^ | .72^***^ | .66^***^ | .18^***^ | .71^***^ | .59^***^ | .43^***^ | .70^***^ | .69^***^ | .63^***^ | .58^***^ | .81^***^ | .67^***^ | .78^***^ | .55^***^ | - |  |
| 1. Story | .69^***^ | .18^***^ | .73^***^ | .38^***^ | .68^***^ | .65^***^ | .59^***^ | .65^***^ | .55^***^ | .74^***^ | .67^***^ | .20^***^ | .73^***^ | .61^***^ | .44^***^ | .69^***^ | .69^***^ | .65^***^ | .60^***^ | .84^***^ | .70^***^ | .80^***^ | .55^***^ | .85^***^ | - |
| 1. Strategy | .69^***^ | .21^***^ | .72^***^ | .37^***^ | .67^***^ | .66^***^ | .64^***^ | .59^***^ | .53^***^ | .75^***^ | .66^***^ | .24^***^ | .74^***^ | .59^***^ | .46^***^ | .61^***^ | .69^***^ | .65^***^ | .62^***^ | .80^***^ | .73^***^ | .73^***^ | .57^***^ | .75^***^ | .79^***^ |

^*^*p* < .05, ^***^*p* < .001

**Supplemental Table S3**

*Factor Loadings Of All GMI Items In Females And Males*

| Motivational factor | Item number | Factor loading | | ΔFactor loading |
| --- | --- | --- | --- | --- |
|  |  | Female | Male |  |
| Advancement | GMI1 | .80 | .81 | .01 |
|  | GMI2 | .94 | .90 | .04 |
|  | GMI3 | .87 | .85 | .02 |
| Amotivation | GMI4 | .87 | .86 | .01 |
|  | GMI5 | .71 | .76 | .05 |
|  | GMI6 | .85 | .76 | .09 |
| Autonomy | GMI7 | .84 | .87 | .03 |
|  | GMI8 | .85 | .85 | .00 |
|  | GMI9 | .89 | .86 | .03 |
|  | GMI10 | .90 | .92 | .02 |
| Boredom | GMI11 | .84 | .84 | .00 |
|  | GMI12 | .84 | .79 | .05 |
|  | GMI13 | .81 | .83 | .02 |
| Competence | GMI14 | .89 | .83 | .06 |
|  | GMI15 | .87 | .84 | .03 |
|  | GMI16 | .91 | .83 | .08 |
| Competition | GMI17 | .85 | .83 | .02 |
|  | GMI18 | .88 | .86 | .02 |
|  | GMI19 | .87 | .89 | .02 |
| Completion | GMI20 | .87 | .85 | .02 |
|  | GMI21 | .93 | .87 | .06 |
|  | GMI22 | .89 | .85 | .04 |
| Coping | GMI23 | .69 | .78 | .09 |
|  | GMI24 | .91 | .91 | .00 |
|  | GMI25 | .87 | .77 | .10 |
| Escape | GMI26 | .91 | .89 | .02 |
|  | GMI27 | .90 | .90 | .00 |
|  | GMI28 | .89 | .85 | .04 |
|  | GMI29 | .86 | .88 | .02 |
| Exploration & Mechanics | GMI30 | .86 | .84 | .02 |
|  | GMI31 | .88 | .88 | .00 |
|  | GMI32 | .92 | .88 | .04 |
|  | GMI33 | .85 | .86 | .01 |
| Fantasy | GMI34 | .88 | .80 | .08 |
|  | GMI35 | .93 | .91 | .02 |
|  | GMI36 | .92 | .87 | .05 |
|  | GMI37 | .80 | .74 | .06 |
| Financial | GMI38 | .95 | .92 | .03 |
|  | GMI39 | .87 | .92 | .05 |
|  | GMI40 | .93 | .92 | .01 |
| Game skills | GMI41 | .91 | .83 | .08 |
|  | GMI42 | .91 | .87 | .04 |
|  | GMI43 | .87 | .90 | .03 |
|  | GMI44 | .91 | .91 | .00 |
| Identity | GMI45 | .84 | .76 | .08 |
|  | GMI46 | .89 | .85 | .04 |
|  | GMI47 | .90 | .87 | .03 |
|  | GMI48 | .88 | .82 | .06 |
|  | GMI49 | .82 | .80 | .02 |
| Introjected regulation | GMI50 | .88 | .87 | .01 |
|  | GMI51 | .92 | .86 | .06 |
|  | GMI52 | .88 | .83 | .05 |
| Recreation | GMI53 | .81 | .83 | .02 |
|  | GMI54 | .85 | .86 | .01 |
|  | GMI55 | .87 | .76 | .11 |
| Skill development | GMI56 | .89 | .87 | .02 |
|  | GMI57 | .89 | .84 | .05 |
|  | GMI58 | .89 | .87 | .02 |
|  | GMI59 | .83 | .83 | .00 |
| Social | GMI60 | .86 | .82 | .04 |
|  | GMI61 | .77 | .75 | .02 |
|  | GMI62 | .88 | .89 | .01 |
|  | GMI63 | .89 | .90 | .01 |
| Status | GMI64 | .88 | .88 | .00 |
|  | GMI65 | .90 | .90 | .00 |
|  | GMI66 | .92 | .87 | .05 |
| Arousal-action | GMI67 | .86 | .84 | .02 |
|  | GMI68 | .88 | .87 | .01 |
|  | GMI69 | .85 | .88 | .03 |
|  | GMI70 | .87 | .85 | .02 |
| Cooperation | GMI71 | .88 | .88 | .00 |
|  | GMI72 | .90 | .90 | .00 |
|  | GMI73 | .92 | .87 | .05 |
| Customization | GMI74 | .90 | .85 | .05 |
|  | GMI75 | .89 | .85 | .04 |
|  | GMI76 | .88 | .88 | .00 |
| Destruction | GMI77 | .84 | .82 | .02 |
|  | GMI78 | .81 | .78 | .03 |
|  | GMI79 | .85 | .80 | .05 |
| Graphics | GMI80 | .76 | .78 | .02 |
|  | GMI81 | .92 | .89 | .03 |
|  | GMI82 | .93 | .91 | .02 |
| Story | GMI83 | .86 | .88 | .02 |
|  | GMI84 | .84 | .87 | .03 |
|  | GMI85 | .86 | .87 | .01 |
| Strategy | GMI86 | .87 | .88 | .01 |
|  | GMI87 | .86 | .86 | .00 |
|  | GMI88 | .89 | .85 | .04 |

**Supplemental Table S4**

*Spearman’s Correlation Coefficients of Direct and Indirect Gaming Involvement with Gaming Motives*

| Zmienna | DGI | IGI |
| --- | --- | --- |
| DGI | - | .70^***^ |
| IGI | .70^***^ | - |
| Advancement | .30^***^ | .33^***^ |
| Amotivation | -.07^*^ | -.03 |
| Autonomy | .30^***^ | .35^***^ |
| Boredom | .16^***^ | .18^***^ |
| Competence | .30^***^ | .35^***^ |
| Competition | .29^***^ | .31^***^ |
| Completion | .26^***^ | .29^***^ |
| Coping | .27^***^ | .31^***^ |
| Escape | .22^***^ | .25^***^ |
| Exploration & Mechanics | .25^***^ | .30^***^ |
| Fantasy | .27^***^ | .31^***^ |
| Financial | -.01 | .01 |
| Game skills | .29^***^ | .34^***^ |
| Identity | .25^***^ | .28^***^ |
| Introjected regulation | .22^***^ | .26^***^ |
| Recreation | .30^***^ | .33^***^ |
| Skill development | .25^***^ | .29^***^ |
| Social | .30^***^ | .36^***^ |
| Status | .27^***^ | .29^***^ |
| Arousal-action | .30^***^ | .34^***^ |
| Cooperation | .29^***^ | .32^***^ |
| Customization | .30^***^ | .33^***^ |
| Destruction | .22^***^ | .26^***^ |
| Graphics | .28^***^ | .32^***^ |
| Story | .27^***^ | .29^***^ |
| Strategy | .25^***^ | .28^***^ |

*Notes:* DGI = Direct gaming involvement; IGI = Indirect gaming involvement; ^*^*p* < .05, ^***^*p* < .001

**Supplemental Table S5**

*Zero-Order Correlation Coefficients of Gaming Disorder and Internet Gaming Disorder with Gaming Motives*

| Zmienna | GDT | IGD |
| --- | --- | --- |
| GDT | - | .91^***^ |
| IGD | .91^***^ | - |
| Advancement | .39^***^ | .40^***^ |
| Amotivation | .35^***^ | .40^***^ |
| Autonomy | .38^***^ | .40^***^ |
| Boredom | .35^***^ | .38^***^ |
| Competence | .45^***^ | .48^***^ |
| Competition | .38^***^ | .39^***^ |
| Completion | .48^***^ | .50^***^ |
| Coping | .46^***^ | .51^***^ |
| Escape | .49^***^ | .57^***^ |
| Exploration & Mechanics | .38^***^ | .43^***^ |
| Fantasy | .45^***^ | .50^***^ |
| Financial | .37^***^ | .42^***^ |
| Game skills | .42^***^ | .45^***^ |
| Identity | .52^***^ | .57^***^ |
| Introjected regulation | .61^***^ | .66^***^ |
| Recreation | .22^***^ | .24^***^ |
| Skill development | .39^***^ | .44^***^ |
| Social | .46^***^ | .49^***^ |
| Status | .50^***^ | .54^***^ |
| Arousal-action | .40^***^ | .43^***^ |
| Cooperation | .36^***^ | .38^***^ |
| Customization | .35^***^ | .35^***^ |
| Destruction | .49^***^ | .50^***^ |
| Graphics | .29^***^ | .31^***^ |
| Story | .30^***^ | .32^***^ |
| Strategy | .32^***^ | .36^***^ |

^***^*p* < .001

**Supplemental Table S6**

*Factor Loadings, Model Fit Indicators And Cronbach's Alpha Values Of The 26 Gaming Motives*

| Motivational factor | Item number | Factor loading | RMSEA | SRMR | CFI | α |
| --- | --- | --- | --- | --- | --- | --- |
| Advancement | GMI1 | .81 | n/a | n/a | n/a | .90 |
|  | GMI2 | .92 |  |  |  |  |
|  | GMI3 | .86 |  |  |  |  |
| Amotivation | GMI4 | .87 | n/a | n/a | n/a | .84 |
|  | GMI5 | .74 |  |  |  |  |
|  | GMI6 | .80 |  |  |  |  |
| Autonomy | GMI7 | .86 | .04 | .01 | 1.00 | .93 |
|  | GMI8 | .86 |  |  |  |  |
|  | GMI9 | .88 |  |  |  |  |
|  | GMI10 | .91 |  |  |  |  |
| Boredom | GMI11 | .84 | n/a | n/a | n/a | .87 |
|  | GMI12 | .82 |  |  |  |  |
|  | GMI13 | .82 |  |  |  |  |
| Competence | GMI14 | .87 | n/a | n/a | n/a | .90 |
|  | GMI15 | .85 |  |  |  |  |
|  | GMI16 | .87 |  |  |  |  |
| Competition | GMI17 | .85 | n/a | n/a | n/a | .90 |
|  | GMI18 | .87 |  |  |  |  |
|  | GMI19 | .88 |  |  |  |  |
| Completion | GMI20 | .86 | n/a | n/a | n/a | .91 |
|  | GMI21 | .90 |  |  |  |  |
|  | GMI22 | .87 |  |  |  |  |
| Coping | GMI23 | .74 | n/a | n/a | n/a | .86 |
|  | GMI24 | .91 |  |  |  |  |
|  | GMI25 | .82 |  |  |  |  |
| Escape | GMI26 | .90 | .08 | .01 | 1.00 | .94 |
|  | GMI27 | .90 |  |  |  |  |
|  | GMI28 | .87 |  |  |  |  |
|  | GMI29 | .87 |  |  |  |  |
| Exploration & Mechanics | GMI30 | .85 | .12 | .01 | .99 | .93 |
|  | GMI31 | .88 |  |  |  |  |
|  | GMI32 | .90 |  |  |  |  |
|  | GMI33 | .86 |  |  |  |  |
| Fantasy | GMI34 | .84 | .05 | .01 | .99 | .92 |
|  | GMI35 | .92 |  |  |  |  |
|  | GMI36 | .89 |  |  |  |  |
|  | GMI37 | .77 |  |  |  |  |
| Financial | GMI38 | .93 | n/a | n/a | n/a | .94 |
|  | GMI39 | .90 |  |  |  |  |
|  | GMI40 | .92 |  |  |  |  |
| Game skills | GMI41 | .87 | <.01 | <.01 | 1.00 | .94 |
|  | GMI42 | .89 |  |  |  |  |
|  | GMI43 | .89 |  |  |  |  |
|  | GMI44 | .91 |  |  |  |  |
| Identity | GMI45 | .81 | .11 | .02 | .99 | .93 |
|  | GMI46 | .87 |  |  |  |  |
|  | GMI47 | .89 |  |  |  |  |
|  | GMI48 | .85 |  |  |  |  |
|  | GMI49 | .81 |  |  |  |  |
| Introjected regulation | GMI50 | .88 | n/a | n/a | n/a | .91 |
|  | GMI51 | .89 |  |  |  |  |
|  | GMI52 | .86 |  |  |  |  |
| Recreation | GMI53 | .82 | n/a | n/a | n/a | .87 |
|  | GMI54 | .85 |  |  |  |  |
|  | GMI55 | .81 |  |  |  |  |
| Skill development | GMI56 | .88 | .14 | .02 | .99 | .92 |
|  | GMI57 | .87 |  |  |  |  |
|  | GMI58 | .88 |  |  |  |  |
|  | GMI59 | .83 |  |  |  |  |
| Social | GMI60 | .84 | .10 | .01 | .99 | .91 |
|  | GMI61 | .77 |  |  |  |  |
|  | GMI62 | .89 |  |  |  |  |
|  | GMI63 | .90 |  |  |  |  |
| Status | GMI64 | .89 | n/a | n/a | n/a | .93 |
|  | GMI65 | .90 |  |  |  |  |
|  | GMI66 | .90 |  |  |  |  |
| Arousal-action | GMI67 | .86 | .12 | .01 | .99 | .92 |
|  | GMI68 | .88 |  |  |  |  |
|  | GMI69 | .86 |  |  |  |  |
|  | GMI70 | .87 |  |  |  |  |
| Cooperation | GMI71 | .85 | n/a | n/a | n/a | .91 |
|  | GMI72 | .89 |  |  |  |  |
|  | GMI73 | .89 |  |  |  |  |
| Customization | GMI74 | .88 | n/a | n/a | n/a | .91 |
|  | GMI75 | .87 |  |  |  |  |
|  | GMI76 | .88 |  |  |  |  |
| Destruction | GMI77 | .84 | n/a | n/a | n/a | .86 |
|  | GMI78 | .80 |  |  |  |  |
|  | GMI79 | .83 |  |  |  |  |
| Graphics | GMI80 | .77 | n/a | n/a | n/a | .90 |
|  | GMI81 | .90 |  |  |  |  |
|  | GMI82 | .92 |  |  |  |  |
| Story | GMI83 | .87 | n/a | n/a | n/a | .90 |
|  | GMI84 | .86 |  |  |  |  |
|  | GMI85 | .87 |  |  |  |  |
| Strategy | GMI86 | .87 | n/a | n/a | n/a | .90 |
|  | GMI87 | .87 |  |  |  |  |
|  | GMI88 | .87 |  |  |  |  |
